# The benefits of more government interference in prescription drug pricing

**DOI:** 10.1016/j.hpopen.2022.100071

**Published:** 2022-04-09

**Authors:** Ryan S. Houser

**Affiliations:** Geogetown University Center for Global Health Science and Security, 3900 Reservoir Road NW, Washington DC, 20057, USA

## Abstract

• Price of prescription drugs reform has gained bipartisan support.
• 25% of Americans find it difficult to afford necessary prescriptions.
• Price caps do not hinder innovation, but rather are common-sense solutions.
• Price caps mandate innovation be conducted in a fiscally responsible and moral way.

The price of prescription drugs is such a large problem that it has gained bipartisan support. While the Trump Administration made strides to improve transparency in pricing through the federal “Transparency in Coverage” rule which requires insurers to disclose list and net prices for medications while also giving patients personalized cost-sharing estimates, enacted with much industry opposition, this action is not a direct measure towards reducing costs. A system that allows lifesaving medication such as epinephrine to cost patients anywhere from $150 to $700 dollars is one that could benefit from prescription drug price control measures [1]. The same is true for insulin in which The United States represents only 15% of the global market, but generates 50% of the revenue [2]. While the net costs to pharmacy benefit managers [PBMs] and payers have decreased over the years, the savings have not been passed to patients who bear major expenses, no matter their insurance coverage [commercial market, Medicare, or un- and underinsured]. The actions proposed by H.R.3 [Lower Drug Costs Now Act] and S.2543 [Prescription Drug Pricing Reduction Act] are necessary to ensure equitable access to lifesaving medications.

H.R. 3 is an important check on the inflated market for prescription drugs. Approximately 25% of Americans find it difficult to afford necessary prescriptions due to the high costs [3]. While the new legislation in Congress is focused on both new and existing drugs, it is particularly important to focus on the costs that are due to existing drugs. In a five year period from 2012 to 2017, the United States spent $6.8 billion due to price increases on existing brand name cancer drugs, while the rest of the world spent $1.7 billion less [4]. The same trend can be seen with insulin in which one vial of Humalog cost $21 in 1999 and now is priced at over $300 [5]. The pharmaceutical industry increased prices on over 250 drugs by 5% on January 1, 2020 [6]. Any measures to control the price increases through government interference should be seen as a benefit to consumers and not a limitation on innovation.

While the Congressional Budget Office (CBO) concludes that there would be approximately 8 fewer drugs introduced into the United States’ market over the next decade and 30 fewer over the subsequent decade, this assumes that pharmaceutical companies would not reduce their costs in other places to allow for more money to invest in research [7]. Individual pharmaceutical companies and their trade organization spent approximately $220 million in lobbying [8]. This accompanied by $30 billion a year in marketing, offers ample opportunity to find investments for research while also having price caps that allow patients to afford the medication. The amount spent on marketing has risen over $12.2 billion in 12 years with direct-to-consumer advertisements for prescription drugs increasing by $7.5 billion in the same time frame. Even with policies at hospitals and medical schools in place to limit pharmaceutical industry influence overprescribing, pharmaceutical marketing to health professionals climbed from $15.6 billion to $20.3 billion [9].

There are no restrictions that are keeping pharmaceutical companies from changing their allocation of revenue to ensure that there continues to be research conducted for new drugs. The fear that “price controls imposed today will result in less research tomorrow” [10] and thus will limit the cures or treatments for various diseases, neglects the ethical and moral responsibility of the pharmaceutical companies to provide innovation for patients in need while also ensuring to not make their medications unobtainable for those most in need. The Biden administration has the opportunity to invest through the Department of Health and Human Services (HHS) more opportunities for research into drug development to also cover any lost jobs that are a concern of price controls [10]. The concern that “restricting or eliminating private sector innovation will leave us far more exposed to emerging threats” [10] ignores the reality of the pandemic where these industries had limited pricing regulations and the United States was still vulnerable to an emerging threat. The private sector provides immense value for the country, especially during public health crises when we are dependent on the strength of our healthcare system. Price caps do not hinder innovation, but rather are common-sense solutions that mandate innovation be conducted in a fiscally responsible and moral way that protects the needs of the American public.

## Declaration of Competing Interest

The authors declare that they have no known competing financial interests or personal relationships that could have appeared to influence the work reported in this paper.
